# DIDA: Distributed Indexing Dispatched Alignment

**DOI:** 10.1371/journal.pone.0126409

**Published:** 2015-04-29

**Authors:** Hamid Mohamadi, Benjamin P Vandervalk, Anthony Raymond, Shaun D Jackman, Justin Chu, Clay P Breshears, Inanc Birol

**Affiliations:** 1 Genome Sciences Centre, British Columbia Cancer Agency, Vancouver, BC, Canada; 2 Department of Bioinformatics, University of British Columbia, Vancouver, BC, Canada; 3 Department of Medical Genetics, University of British Columbia, Vancouver, BC, Canada; 4 School of Computing Science, Simon Fraser University, Burnaby, BC, Canada; 5 Intel Health and Life Sciences, Intel Corporation, Hillsboro, OR, US; Oak Ridge National Lab, UNITED STATES

## Abstract

One essential application in bioinformatics that is affected by the high-throughput sequencing data deluge is the sequence alignment problem, where nucleotide or amino acid sequences are queried against targets to find regions of close similarity. When queries are too many and/or targets are too large, the alignment process becomes computationally challenging. This is usually addressed by preprocessing techniques, where the queries and/or targets are indexed for easy access while searching for matches. When the target is static, such as in an established reference genome, the cost of indexing is amortized by reusing the generated index. However, when the targets are non-static, such as contigs in the intermediate steps of a *de novo* assembly process, a new index must be computed for each run. To address such scalability problems, we present DIDA, a novel framework that distributes the indexing and alignment tasks into smaller subtasks over a cluster of compute nodes. It provides a workflow beyond the common practice of embarrassingly parallel implementations. DIDA is a cost-effective, scalable and modular framework for the sequence alignment problem in terms of memory usage and runtime. It can be employed in large-scale alignments to draft genomes and intermediate stages of *de novo* assembly runs. The DIDA source code, sample files and user manual are available through http://www.bcgsc.ca/platform/bioinfo/software/dida. The software is released under the British Columbia Cancer Agency License (BCCA), and is free for academic use.

## Introduction

Performing fast and accurate alignments of reads generated by modern sequencing technologies represents an active field of research. At its core, the sequence alignment problem is about identifying regions of close similarity between a query and a target. Most modern algorithms in this domain work by first constructing an index of the target and/or the query sequences. This index may be in the form of a suffix tree [1, 2], suffix array [3, 4], hash table [5–13], or full-text minute-space index (FM-index) [14–20]. Although this pre-processing step introduces an initial computational overhead, indexing helps narrow the list of possible alignment coordinates, speeding up the alignment task.

When the target sequence is static (e.g., a reference genome), the cost of index construction represents a one-time fixed-cost. It is performed once as a pre-alignment operation, and the resulting index is used for many subsequent queries. Hence, it is often discounted in performance measurements of alignment tools. However, there are many applications where the reference is not static and/or the computational cost of indexing is not negligible. Such cases include resequencing data analysis of non-model species, where the target index has to be established, and intermediate stages of a *de novo* assembly process where index construction needs to be performed several times.

One more complicating factor in these two domains is that the target sequence may not represent chromosome-level contiguity, requiring alignments to a fragmented target sequence. This may be a particular challenge for many alignment algorithms, which perform poorly near target boundaries, introducing “edge effects”.

To address these challenges, we have designed and developed DIDA, for Distributed Indexing and Dispatched Alignment. DIDA works by first distributing the index construction over several computing nodes. It dispatches the query sequences over corresponding computing nodes for alignment. Finally, partial alignment results from different computing nodes are gathered and combined for reporting.

We tested DIDA using four datasets: (1) *C. elegans* genome, (2) Human draft genome, (3) Human reference genome, and (4) *P. glauca* genome. Here, we report on the scalability, modularity and performance of the tool.

## Materials and Methods

The sequence alignment task is often suitable for parallel processing, and the widely practiced approach is to perform the task in an embarrassingly parallel manner. When the target index is available, it is loaded on multiple processors, and a subset of the query sequences (usually raw reads from a sequencing experiment) are aligned in parallel to this common target.

**Fig 1:**
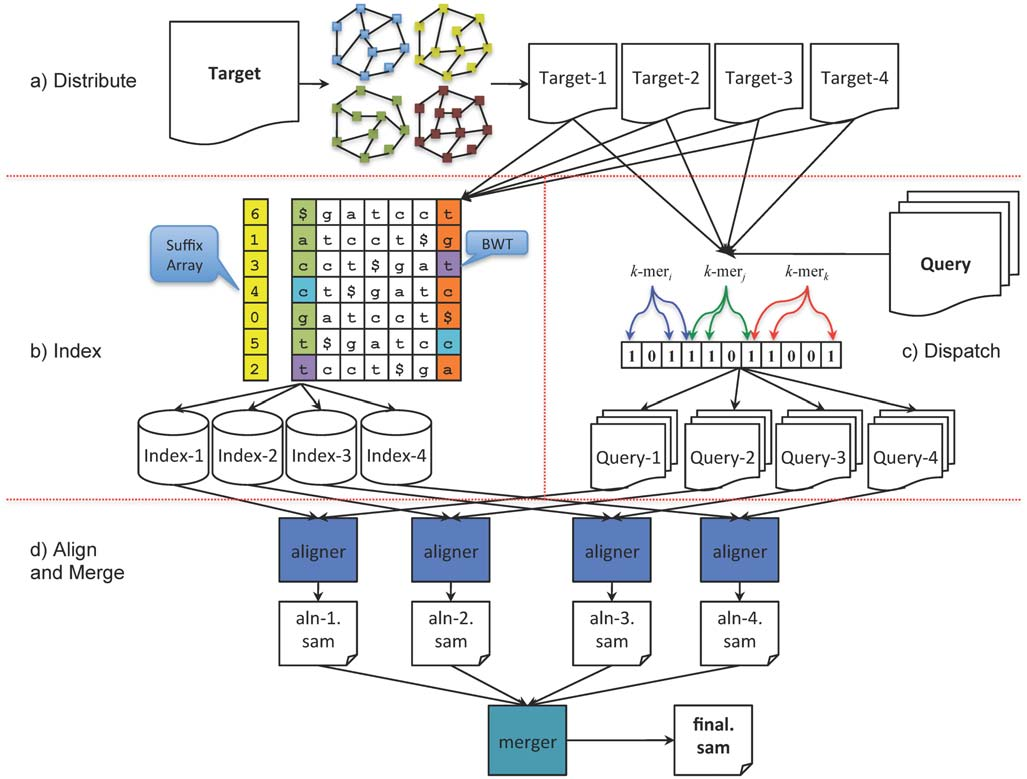
DIDA workflow with four partitions as an example. (a) First, we partition the targets into four parts using a heuristic balanced cut. (b) Next, we create an index for each partition. (c) The reads are then flowed through Bloom filters to dispatch the alignment task to the corresponding node(s). (d) Finally, the reads are aligned on all four partitions and the results are combined together to create the final output.

In DIDA, we parallelize both the indexing and alignment operations using a five-step workflow (Fig 1 and S1 Table). For the description of the method, we consider a use case where the target is a draft genome assembly, with individual contigs and scaffolds related to each other through an assembly graph. Although, the protocol is general enough for a generic target not associated with a graph. Before describing the proposed framework, we provide some preliminary and basic definitions and notation.

### Assembly Graph

Most modern assembly tools are graph-based algorithms [18, 21–25]. These algorithms model the assembly problem using a graph data structure (e.g., *de Bruijn* graph, overlap graph, string graph) consisting of a set of vertices (reads or *k*-mers) and edges (overlaps) representing the relationship between vertices. After building such graphs, the assembly problem is converted to a graph traversal problem, where a walk along the graph would reconstruct the source genome. In practice, assembly algorithms report unambiguous walks on the assembly graphs, building contigs as opposed to full genomes or chromosomes. Especially for large genomes, use of short reads from high-throughput sequencing platforms for assembly results in a large number of contigs. For example, using 150 base pairs (bp) reads, the white spruce genome is assembled into several million contigs [26].

Some of the ambiguity on the assembly graph can be mitigated by using paired end reads or other linkage information. This requires alignment of queries to a typically highly fragmented draft genome. In DIDA, when available, we partition the assembly graph keeping tightly connected components on the same partition, as described below. For other use cases, where the target components (e.g., contigs or chromosomes) are not related through a graph, the partition optimization is done based on component lengths.

### Bloom filter

A Bloom filter [27] is a compact and space-efficient probabilistic data structure providing membership queries over dynamic sets with an allowable false positive rate. It has been widely used in many computing applications, which exploit its ability to succinctly represent a set, and efficiently filter out items that do not belong to the set, with an adjustable error probability. In bioinformatics, the Bloom filter has been recently utilized in applications such as *k*-mer counting, genome assembly and contamination detection [28–32].

An ordinary Bloom filter consists of a bit array *B* of *m* bits, which are initially set to 0, and *k* hash functions, *h*_1_, *h*_2_, …, *h*_*k*_, mapping keys from a universe *U* to the bit array range {1, 2, …, *m*}. In order to insert an element *x* from a set *S* = {*x*_1_, *x*_2_, …, *x*_*n*_} to the filter, the bits at positions *h*_1_(*x*), *h*_2_(*x*), …, *h*_*k*_(*x*) are set to 1. To query if an element *q* is in the filter, all of the bits at positions *h*_1_(*q*), *h*_2_(*q*), …, *h*_*k*_(*q*) are examined. If at least one bit is equal to 0, then *q* is definitely not in *S*. Otherwise, *q* likely belongs to the set. The uncertainty stems from coincidental cases, where all the corresponding bits, *h*_*i*_(*q*) *i* = 1, 2, …, *k*, may be equal to one even though *q* is not in *S*. This is possible if other keys from *S* were mapped into these positions. Such a chance occurrence is called a false positive hit, and its probability is called the false positive rate, *F*. The probability for a false positive hit depends on the selection of the parameters *m* and *k*, the size of the bit array and the number of hash functions, respectively. After inserting *n* distinct elements at random to the bit array of size *m*, the probability that a specific bit in the filter is 0 is ${\left(1-\frac{1}{m}\right)}^{kn}$. Therefore, the false positive rate is:

$$\begin{array}{c} F={\left(1-{\left(1-\frac{1}{m}\right)}^{kn}\right)}^{k}\approx {\left(1-e^{-k/r}\right)}^{k} \end{array}$$ (1)

where *r* = *m*/*n* is the number of bits per element. Minimizing the Eq (1) for a fixed ratio of *r* yields the optimal number of hash functions of *k* = *r*ln(2), in which case the false positive rate is (0.6185)^*r*^ [33].

### DIDA

Our proposed distributed and parallel indexing and alignment framework, DIDA, consists of five major steps to perform the indexing and alignment task: distribute, index, dispatch, align, and merge. The indexing and dispatch steps are performed in parallel. Each step is explained in detail as follows.

**Distribute.** In this step, the set of target sequences is partitioned into several subsets. Depending on the nature of the target sequences (static as in reference genomes, or non-static as in a draft assembly; unrelated as in chromosomes, or related as contigs in an assembly graph) different partitioning strategies may apply to initial target set. The key point in all cases is to keep the partitions as balanced as possible. The target partitioning problem is a variant of the bin-packing problem and since the bin-packing problem is NP-hard, there is no polynomial time solution to the target partitioning problem either. However, there are efficient heuristics developed to solve the problem [34, 35]. Other than the theoretical hardness of the target partitioning problem, having well-balanced partitions in practice when the target set contains few number of long sequences will also be difficult.

In the case of a static and independent set of target sequences, the partitioning is performed using the best fit decreasing strategy that is among the simplest greedy approximation algorithms for solving the bin-packing problem. Here, the bins correspond to computing nodes, and items are the target sequences. This strategy operates by first sorting the target sequences to be partitioned in decreasing order by their lengths, and then distributing each target sequence into the best node in the list, which is the node with the minimum sufficient remaining space for the target sequence.

When the target sequences are related, such as contigs in a draft assembly, the partitioning starts by first identifying all connected target sequences using adjacency information in the assembly graph. This is performed by launching a depth-first search traversal of the undirected adjacency graph, and by finding all connected components. Then, the partitioning procedure continues by applying the best fit decreasing strategy for identified connected components to distribute them over computing nodes.

**Index.** An exact pattern search can take linear time in the target length. But when the target length is very long, it is desirable to have the search time linear in the query length and independent of the target length. To do so, an index such as a suffix tree, suffix array [36–38], hash table, or FM-index [39, 40] on the long sequence or text can be created. Constructing such an index takes *O*(*n*) time and *O*(*n*log*n*) space, where *n* is the size of the target [38]. This cost is often amortized when the index is used several times, providing very fast searching of the indexed target.

For the indexing step, and on each computing node, DIDA takes the subset of target sequences from the Distribute step, and constructs an index for each subset in parallel on all computing nodes. Then, the indices are stored on each computing node to be invoked later in the alignment step. Depending on the alignment algorithm, any indexing approach can be used in this step. The reduced target size (*n*/*P* in the best-case scenario, where *P* is the number of partitions) allows linear scaling of the indexing time and better than linear scaling in the index space. While both would have a positive impact on alignments against dynamic targets, the latter would also help cases where the target is too large to fit into the memory of a single computer.

**Dispatch.** To keep track of each subset of target sequences, and to identify which read may align on which partition of the target, a set of Bloom filters is created for all partitions of target sequences. The reads are then *flowed* through these Bloom filters, and dispatched to the corresponding node(s).

To create a Bloom filter for each partition of target sequences, all target subsequences of length *b* (*b*-mer) in the partition are scanned. Each scanned *b*-mer, *x*, is then inserted into the corresponding Bloom filter by setting the related bits in the bit array, i.e. *B*[*h*_*i*_[*x*]] = 1, *i* = 1, 2, …, *k*. After constructing the Bloom filters, all possible read *b*-mers are queried against the Bloom filters. If at least one hit is found for each read, the read is dispatched to the corresponding node. This procedure continues until all the reads are either dispatched or discarded. By choosing the *b*-mer length, *b*, small enough, we make sure that no read sequence will be missed in the Bloom filter query step. This is performed by setting *b* less or equal than the minimum seed or exact match length of aligners, *l*, for candidate hits. Detailed procedure for choosing *b*, loading and querying Bloom filters is presented in S1 Text.

In the implementation, the values of *r* and *k* can be set as input parameters and we have considered 8 bits for each *b*-mer, *r* = 8, as default. Therefore, the optimal number of hash functions that minimizes the false positive rate of Bloom filter is *k* = *r*ln2 ≈ 5, resulting in a false positive error rate slightly larger than 2%. It should be mentioned that the false positive rate does not affect the final alignment result. It only imposes more workload on nodes by dispatching reads that do not necessarily belong to those nodes as a result of false positive Bloom filter hits. S1 Fig shows an example of how different values of *r* and *k* affect the number of extra dispatched reads over multiple nodes.

**Align and Merge.** After constructing indices for all sets of target sequences and dispatching the reads to the computing nodes, DIDA aligns the reads against the target sequence in parallel on each node. Note that, DIDA itself does not offer an alignment algorithm; instead, it can use a variety of third party alignment tools.

In the merging step, partial alignment results, usually stored as SAM/BAM [17] records from different computing nodes, are gathered and combined into the final SAM/BAM output. Depending on the aligner parameters for reporting the output, different merging approaches are applied. For example, when aligner parameters are adjusted in order to obtain the best unique mapped query, the merger will take into account that information to pick up the best quality mapped record for each query from the related records in all partitions. With the reporting parameters set to obtain multiple alignment records, the merger procedure searches for all or up to a predefined distinct number of alignment records in the partial alignment results in all partitions.

The workflow and algorithm of DIDA are presented in Fig 1 and S1 Table.

### Implementation

DIDA is written in C++ and parallelized using OpenMP for multi-threaded computing on a single computing node. For distributed computing, DIDA employs Message Passing Interface (MPI) for inter-process communications. As input, it gets the set of target sequences and the set of queries in FASTA or FASTQ formats, and the default output alignment format is SAM (Sequence Alignment/Map Format).

### Evaluated tools

To evaluate the performance of DIDA, four alignment tools have been used within the proposed framework: BWA-MEM [41], Bowtie2 [15], Novoalign (http://www.novocraft.com), and ABySS-map [24]. A summarized description of each alignment method along with its indexing approach is presented below.

BWA is an FM-index based aligner. It starts by creating an index for target sequences to find exact matches. For inexact matches, it employs a backtracking strategy; within a defined distance, it looks for matches between a substring of target sequences and the read sequence.

Bowtie2 is also an FM-index based alignment method. After constructing the index for target sequences, it uses a modified Ferragina and Manzini [40] matching algorithm to identify the possible mapping coordinates. For inexact matches, it extends the exact match technique with a quality-aware backtracking algorithm that permits mismatches.

Novoalign is a hash-based alignment method. It builds a hash table by dividing the target sequences into overlapping *k*-mers. In the mapping phase, it utilizes the Needleman-Wunsch dynamic programming algorithm [42] with affine gap penalties to find the optimal global alignment.

Similar to BWA and Bowtie2, ABySS-map, a utility within the ABySS genome assembly software [24], constructs an FM-index for target sequences to perform exact matches. It is mainly used for alignment tasks in the intermediate stages of the ABySS assembly pipeline. In order to speed up the alignment operations, and hence the total assembly process, ABySS-map only performs exact matching and avoids backtracking for inexact matching.

All four tools are run with their default parameters, and the parameters related to the resource usage are set in a way to utilize the maximum capacity on each computing node as described in S2 Text. For example, all tools are run in multi-threaded mode with the maximum number of threads on each node. The performance of each alignment method is compared with itself within the DIDA framework.

Results were obtained on a high performance computer cluster consisting of 500 computing nodes, each of which has 48 GB of RAM and dual Intel Xeon X5650 2.66GHz CPUs with 12 cores. The operating system on each node is Linux CentOS 5.4. The cluster’s network fabric and file system are Infiniband 40 Gbps and the IBM GPFS, respectively.

## Results

### Performance on real data

To evaluate the performance and scalability of DIDA on real data, we downloaded publicly available sequencing data on *C. elegans* genome, Human draft assembly, Human reference genome, and *P. glauca* (white spruce) genome from the following websites.

- *C. elegans*: http://www.ncbi.nlm.nih.gov/sra/?term=ERR294494
- Human genome (NA12878): http://www.nature.com/ng/journal/v43/n5/full/ng.806.html
- Human genome reference (hg19, GRCh37): http://hgdownload.cse.ucsc.edu/goldenPath/hg19/database/
- *P. glauca* (accession number: ALWZ0100000000 and PID: PRJNA83435): http:/www.ncbi.nlm.nih.gov/bioproject/83435

In order to assess the performance of DIDA for each aligner on non-static targets, we assembled the reads from each dataset using ABySS 1.3.7, and used the assembly graph in intermediate stages to guide partitioning. We have also evaluated the performance of DIDA for each aligner on human genome reference (hg19, GRCh37) as a static target. The detailed information of each dataset is presented in Table 1.

**Table 1:** Dataset specification.

Data	#targets	target(bp) length	#reads	read(bp) length
*C. elegans*	152,841	106,775,302	89,350,844	8,935,084,400
Human	6,020,169	3,099,949,065	1,221,224,906	123,343,715,506
hg19	93	3,137,161,264	1,221,224,906	123,343,715,506
*P. glauca*	70,868,549	35,816,518,982	1,079,576,520	161,936,478,000

**Fig 2:**
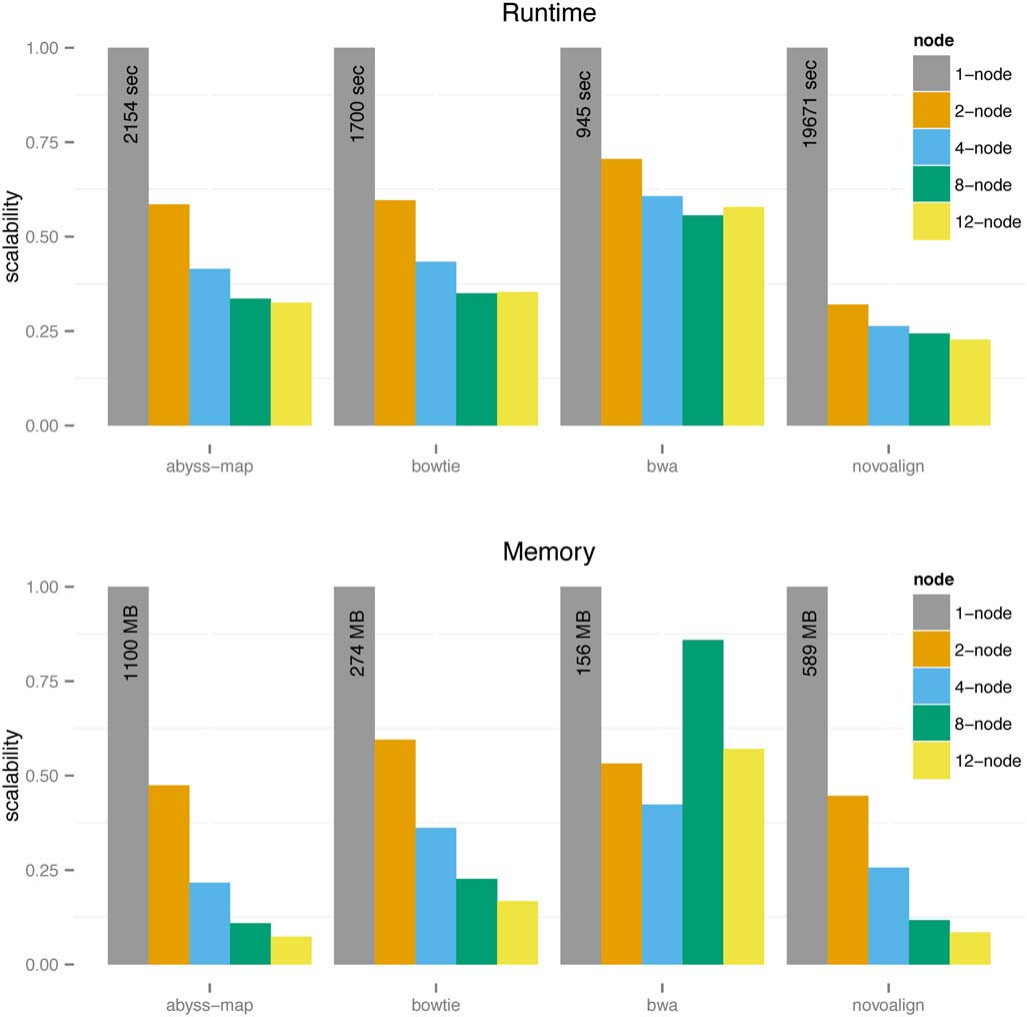
Scalability of different aligners using DIDA for *C. elegant* data. Y-axis indicates the runtime/memory scalability in the in the [0.1] interval for different alignment tools. The scalability of each tool is shown in the standalone case and within DIDA framework on 2, 4, 8, and 12 nodes.

Fig 2 shows the scalability of wall-clock time and indexing peak memory usage of all four aligners on the *C. elegans* dataset, in standalone case (grey bars) or within the DIDA framework on two, four, eight, and 12 nodes, as indicated. For instance, for ABySS-map, the runtime of 2154 sec without DIDA is decreased to 893 sec using DIDA on four computing nodes. From Fig 2 we can see the runtime scalability and modularity of different aligners within DIDA protocol. Notably, we have better scalability for the slower yet more sensitive alignment tool, Novoalign. On memory usage, all aligners scale well within the DIDA framework. For example, the peak memory usage of ABySS-map indexing goes from 1100 MB without DIDA to 238 MB with DIDA on four computing nodes. Detailed information related to runtime and memory usage for all datasets are presented in S2–S5 Tables and Table 2 summarizes all results in the form of alignment-time/indexing-memory. Regarding the accuracy of the alignment results for all aligners within DIDA framework on multiple nodes, we have compared them with the baseline results and found the accuracy of results the same as expected. As mentioned in the previous section, by choosing the *b*-mer length value small enough, we make sure that no potential alignment is missed in the dispatch step, and therefore, the accuracy of final alignment results will not be degraded (S1 Text). For example, the number of aligned reads for total 89,350,844 reads in the *C. elegans* dataset using ABySS-map within DIDA on 2, 4, 8, and 12 nodes is 86,851,694 which is the same as in baseline or standalone mode with the same SAM/BAM quality scores.

**Table 2:** Alignment time/indexing memory for all aligners on different datasets.

*C. elegans* (sec/MB)				
	abyss-map	bwa	bowtie	novoalign
1-node	2154/1100	945/156	1700/274	19671/589
2-node	1261/522	667/80	1014/163	6305/263
4-node	893/238	574/65	737/99	5184/151
8-node	723/120	526/134	595/62	4788/69
12-node	700/81	547/89	601/46	4464/50
human draft assembly (min/MB)				
	abyss-map	bwa	bowtie	novoalign
1-node	652/31000	407/4400	1174/6100	59125/9300
2-node	472/15000	254/2200	611/2900	35728/4100
4-node	343/8100	216/1100	493/1600	23311/3700
8-node	253/4100	191/559	371/977	17485/2100
12-node	210/2700	181/372	296/590	13141/1200
hg19 (min/MB)				
	abyss-map	bwa	bowtie	novoalign
1-node	444/33823	379/4709	996/5528	NA
2-node	323/16911	254/2354	512/3042	NA
4-node	232/8455	205/1177	352/1417	NA
8-node	173/4227	171/588	254/667	NA
12-node	160/3170	164/441	226/495	NA
*Picea glauca* (min/GB)				
	abyss-map	bwa	bowtie	novoalign
1-node	NA	NA	NA	NA
2-node	1201/184	NA	NA	NA
4-node	827/81	NA	NA	NA
8-node	638/45	NA	NA	NA
12-node	574/31	NA	NA	NA

One point that should be addressed is that by increasing the computational power, *i.e.*, number of computing nodes, we may not necessarily obtain better runtime scalability (S3 Text). For instance, the runtime of Bowtie2 within DIDA framework on eight computing nodes is 595 sec compared to 601 sec on 12 computing nodes. This is because of the related overhead of the dispatch and merge steps. Another point that should be explained is the unexpected memory scalability for BWA from 4 to 8 nodes on the *C. elegans* dataset. Based on the size of target set, bwa-index automatically chooses between *bwtsw* and *is* (induced sorting) algorithms to generate BWT (Burrows Wheeler Transform) in the index construction process. For short target sets (≤ 25Mb), bwa-index uses *is* algorithm while for long target sets (> 25Mb) it employs *bwtsw*. The memory usage of *bwtsw* is less than *is* for a given target set. When we divide *C. elegans* dataset into 8 or more partitions, the size of each subset will be less than 25Mb, and hence, bwa-index automatically invokes *is* algorithm. On the other hand, for 4 partitions or less, bwa-index uses *bwtsw* algorithm. Therefore, we see the memory scalability of BWA for *C. elegans* is not as expected.

**Fig 3:**
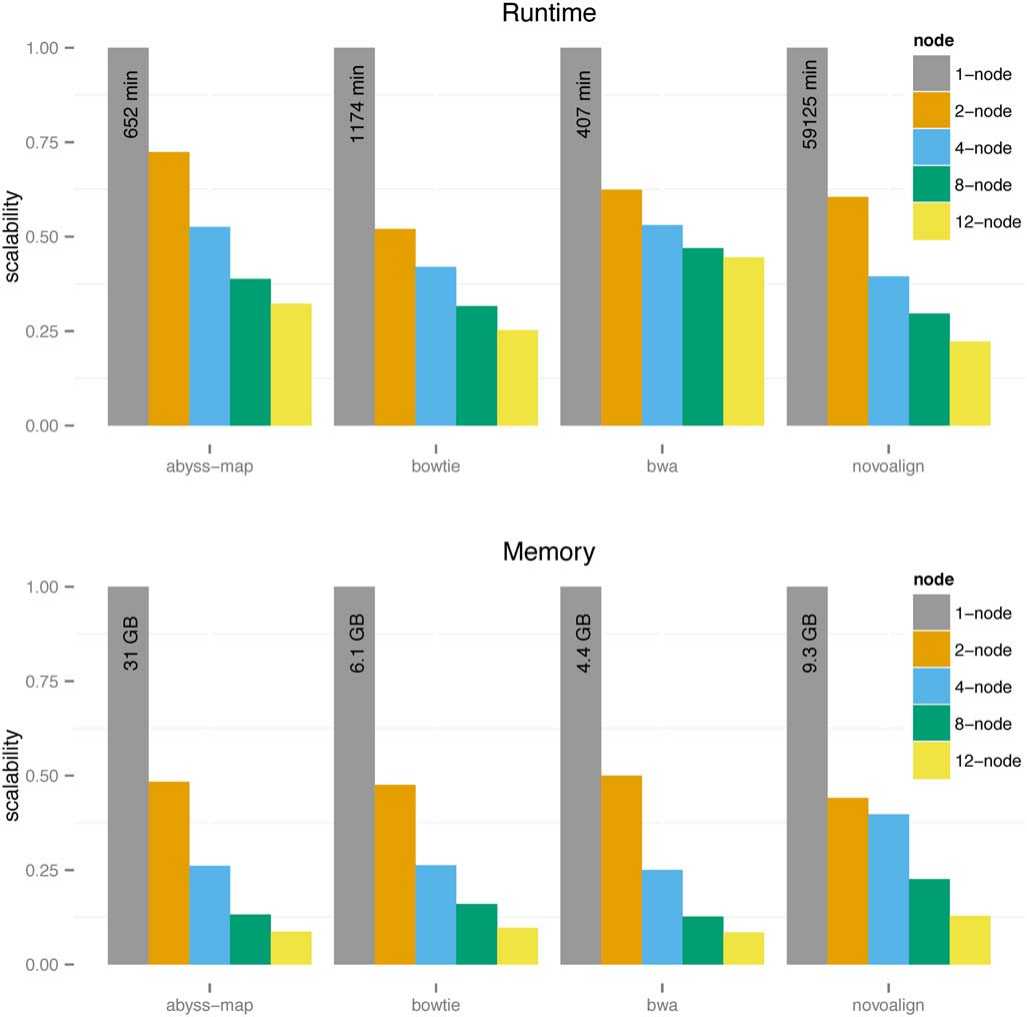
Scalability of different aligners using DIDA for human draft assembly.

Fig 3 shows the result on the human draft assembly data. Compared to the smaller datasets, for human genome we see better runtime and memory usage scalability, illustrating that DIDA shows better performance on large data due to the overhead of distributed paradigm. That means the overhead of dispatch and merge steps are compensated for large-scale indexing and alignment applications. We have also evaluated the performance of DIDA on the human reference genome (hg19) as a static target set. Table 2 shows the scalability of wall-clock time and indexing peak memory usage of different aligners, except Novoalign (due to its long runtime). As expected, the scalabilities for runtime and memory are similar to the case of non-static target set.

Table 2 shows the result for ABySS-map on *P. glauca* draft assembly. Due to resource restrictions, we could not obtain the result of ABySS-map aligner without DIDA. The required memory for constructing the index for the spruce draft assembly is about 400 GB. However, using DIDA framework we can divide the draft spruce assembly into a number of partitions, and perform the indexing and alignment operation in a distributed way. From Table 2, we can easily see the scalability for runtime and indexing peak memory usage of ABySS-map within DIDA.

## Discussion

Indexing large target sequences, and aligning large queries are computationally challenging. In this article, we described a novel, scalable and cost-effective parallel workflow for indexing and alignment, called DIDA.

The performance of DIDA was measured and evaluated when coupled with popular alignment methods BWA, Bowtie2, Novoalign, and ABySS-map on *C. elegans*, human draft genome, human reference genome, and *P. glauca* genome. Compared to their baseline performance, when run through the DIDA framework with 12 nodes, BWA, Bowtie2, Novoalign, and ABySS-map use less memory (91%, 90%, 87%, and 91%, respectively) and execute faster (55%, 74%, 77%, and 67%, respectively) for a draft human genome assembly.

DIDA is an enabling technology for labs that have limited compute resources. For example, for the *P. glauca* draft genome [26], the required memory for index construction on a single node is about 400 GB of RAM, which requires the use of a special big memory machine, and may be prohibitive for many labs. Using the DIDA framework, the indexing was performed in a distributed way on 12 low-memory compute nodes with peak memory usage of 31 GB on any one node. Therefore, DIDA efficiently made this huge indexing and alignment task feasible, well scalable, and modular. This enabled our lab to perform many experiments at a time without requisitioning large memory machines.

As the cost of DNA sequencing is dropping faster than the cost of computational power, the need for scalable and cost-effective parallel and distributed algorithms and software tools for accurately and expeditiously processing “big data” from high-throughput sequencing is increasing. DIDA offers a solution to this growing issue for the alignment problem, especially when the target is non-static, or large. In life sciences research organizations and clinical genomics laboratories, alignment and *de novo* assembly are becoming two key steps in everyday research and analysis. Since many labs may have limited computational resources, DIDA may be an appropriate solution to address their needs and expectations by reducing heavy computational resource requirements.

## Supporting Information

S1 Fig Extra dispatched reads vs. *r* and *k*. (PDF)

S1 Text Procedure for choosing *b*-mer length, BF loading, and querying. (PDF)

S2 Text Command details for running different programs. (PDF)

S3 Text Runtime scalability behaviour. (PDF)

S1 Table DIDA algorithm pseudo code. (PDF)

S2 Table Exact numbers for *C. elegans* dataset—Fig 2 in main text. (PDF)

S3 Table Exact numbers for human draft assembly dataset—Fig 3 in main text. (PDF)

S4 Table Exact numbers for human reference genome (hg19) dataset. (PDF)

S5 Table Exact numbers for *Picea glauca* dataset. (PDF)
