# Supplementary material for: DIDA: Distributed Indexing Dispatched Alignment
Source: PLoS One. 2015 Apr 29;10(4):e0126409. doi: 10.1371/journal.pone.0126409 (PMC4414605; doi:10.1371/journal.pone.0126409)
Supplement: S1 Fig — (PDF) [file pone.0126409.s001.pdf]

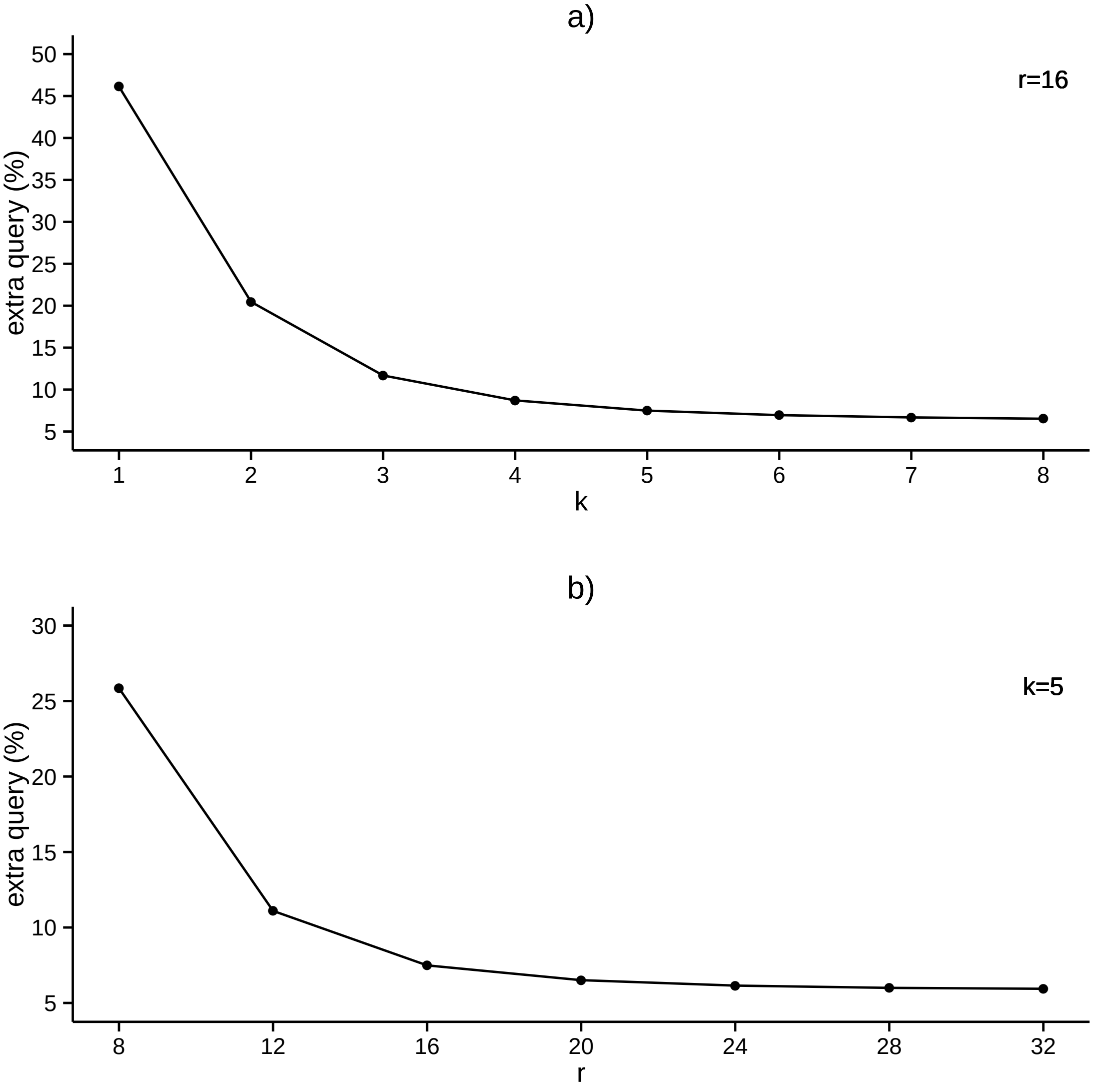


**Supplementary Figure 1.** Extra dispatched reads vs. $r$ and $k$. (a) Percentage of extra dispatched reads vs. the number of hash functions, $k$, for the fixed value of $r = 16$ on *C. elegans* dataset. (b) Percentage of extra dispatched reads vs. the number of bits per item, $r$, for the fixed value of $k = 5$ on *C. elegans* dataset.
