# Supplementary material for: DIDA: Distributed Indexing Dispatched Alignment
Source: PLoS One. 2015 Apr 29;10(4):e0126409. doi: 10.1371/journal.pone.0126409 (PMC4414605; doi:10.1371/journal.pone.0126409)

Supplementary Text 1. Procedure for choosing b-mer length, and BF loading and querying

Loading Bloom filter. In this stage, all target sequences in the target set $T = \{T_1, T_2, \ldots\}$ are scanned using a sliding substring of length b which is called b-mer. For each target sequence T_i, all possible $|T_i| - b + 1$ b-mers are scanned and then inserted to the Bloom filter after specifying the corresponding bit vector positions by computing the k hash values for each b-mer.

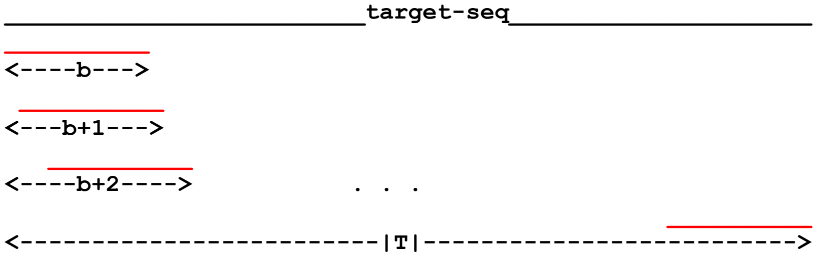

Querying Bloom filter. In this step, all reads in the read set $R = \{R_1, R_2, \ldots\}$ are queried using the same b-mer in the loading stage. If at least one hit is found for a read, the read is dispatched to the corresponding node. We can use sliding b-mer windows (with step size of one base, $s = 1$) or jumping b-mer windows (with step size greater than one base) to interrogate each read R_j as explained below.

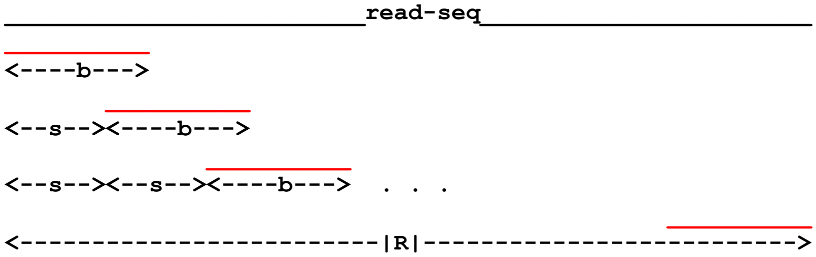

Suppose that the minimum seed or exact match length to report a candidate hit for an aligner is l. We choose $b \leq l$ and then load the Bloom filters as mentioned in the *Loading Bloom filter* stage. In the querying stage, the b-mers of each read sequence is interrogated against all Bloom filters from different partitions. If $b = l$, the reads are scanned using sliding window with step size of one base, i.e. $s = 1$. By choosing $b < l$, the interrogation step will be faster but with more extra dispatched reads, and the step size is computed as follows to cover all possible seeds or exact matches of length l between the target sequence T_i and read sequence R_j . If the l-mer starting at position p is covered by at least one b-mer, to cover the next l-mer starting at position $p + 1$ we should have $s + b \leq l + 1$. Therefore, $s \leq l - b + 1$.

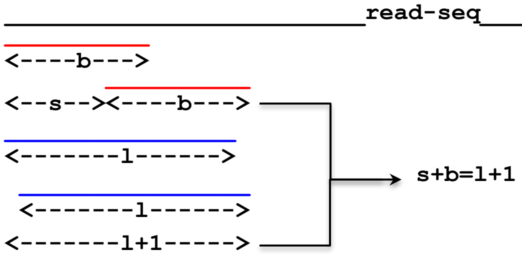

Supplement: S1 Text — (PDF) [file pone.0126409.s002.pdf]
