# Supplementary material for: DIDA: Distributed Indexing Dispatched Alignment
Source: PLoS One. 2015 Apr 29;10(4):e0126409. doi: 10.1371/journal.pone.0126409 (PMC4414605; doi:10.1371/journal.pone.0126409)
Supplement: S2 Text — (PDF) [file pone.0126409.s003.pdf]

Supplementary Text 2. Command details for running the programs

DIDA batch version. To align a library of read files named *query.in* against the target named *target* using the batch version of DIDA, run the set of following commands:

1. prt -p <partition> <target>
2. index <sub-target>
3. dsp [options] -p <partition> -l <l-mer> <query.in>
4. map <sub-query> <sub-target>
5. mrg -p <partition> -a <aligner> -m <mode>

In *dsp* command, -l <l-mer> is the minimum seed or exact match length related to aligners for candidate hits. [options] are:

- -b: specifies the b-mer length for loading and querying Bloom filters [$3l/4$].
- -s: specifies the step size when breaking a query sequence into b-mers.
- -h: specifies the number of hash functions for Bloom filter, [default=5].
- -i: specifies the number of bits per item for Bloom filter, [default=8].

In *mrg* command, <mode> values are:

- mem: this mode is coupled with -r <record> option and reports the best alignment records for each read sequence up to the number specified by -r <record> option. The default value for -r <record> is [5].
- best: the final alignment result includes the best quality mapped record for each read sequence from the related records in all partitions.
- ord: reports all alignment records for each read sequence with the output order matching order of input reads.
- fast: merges all partial alignment results from different partitions very quickly without keeping the order of input reads.

In the command lines 2 and 4, instead of *index <sub-target>* and *map <sub-query> <sub-target>*, different indexing and alignment commands related to each method can be used which are as follows:

- BWA - version 0.7.10
  1. bwa index <target>
  2. bwa mem -k <k-mer> -t <max−thread> <target> <query>
- Bowtie2 - version 2.1.0
  1. bowtie2-build <target> <target-index>
  2. bowtie2-align --reorder -p <max−thread> -x <target-index> -U <query>
- Novoalign - version 3.01.02
  1. novoindex -k <k-mer> <target-index> <target>

    2. novoalign -o Sync -o SAM -f <query> -d <target-index>

- ABySS-map - version 1.5.2

    1. abyss-index <target>
    2. abyss-map -l <*k*-mer> -j <max−thread> --order <query> <target>

DIDA wrapper and mpi versions. In the wrapper version of DIDA, dida-wrapper, all steps in the batch version are linked together using Message Passing Interface (MPI). dida-wrapper can be further streamlined through implementing a massage passing interface protocol that sends reads or alignment records directly to the corresponding nodes instead of using temporary files to dispatch reads to target partitions or alignment record to the merger. This fully-streamlined version of DIDA is named dida-mpi.

To align a library of reads files named *query.in*={qfile1,qfile2,...} against the target set named *target* using the dida-wrapper or dida-mpi versions on n partitions use:

- mpirun -np <n+1> dida-wrapper [options] -l <l-mer> -a <aligner> <qfile1,qfile2,...> <target>
- mpirun -np <n+2> dida-mpi [options] -l <l-mer> -a <aligner> <qfile1,qfile2,...> <target>

Options for dida-wrapper and dida-mpi versions are similar to the batch version.
