# Supplementary material for: DIDA: Distributed Indexing Dispatched Alignment
Source: PLoS One. 2015 Apr 29;10(4):e0126409. doi: 10.1371/journal.pone.0126409 (PMC4414605; doi:10.1371/journal.pone.0126409)
Supplement: S3 Text — (PDF) [file pone.0126409.s004.pdf]

Supplementary Text 3. Runtime scalability behaviour

By increasing the computational power, *i.e.*, number of computing nodes, we may not necessarily obtain better runtime scalability due to the related overhead of the dispatch and merge steps. In general, for any parallel algorithm the runtime scalability should follow the behaviour shown in the following figure.

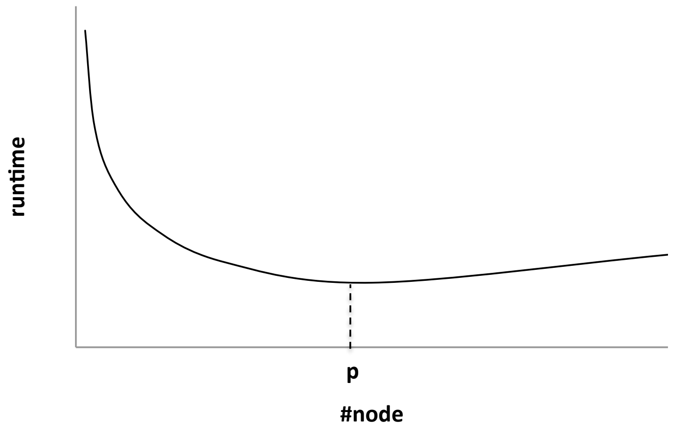

The optimal value for the number of nodes, p, may be different for each dataset and each algorithm. For example, for a small dataset such as *C. elegans*, with the BWA and Bowtie2 aligners, p is between 8 to 12 nodes. For ABySS-map and Novoalign we have $p > 12$. The scalability performance of DIDA for *C. elegans* dataset and ABySS-map alignment method for [2, 4, 8, 16, 32] nodes is presented in the following figure.

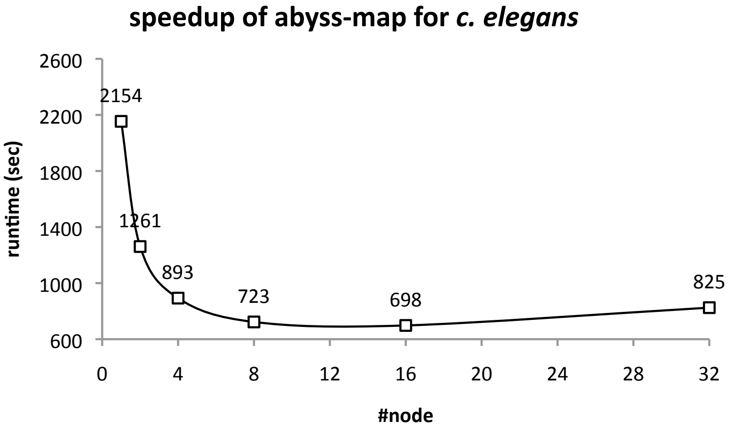

For larger datasets such as human genome, the value of p is greater than 12. In general, we have the fact that the larger the datasets or the faster the alignment methods, the greater the value of p. Since the maximum number of cores on each node in our compute cluster was 12, we chose the maximum value of 12 for the number of partitions resulting in the best performance for dispatch step.
