# Supplementary material for: DIDA: Distributed Indexing Dispatched Alignment
Source: PLoS One. 2015 Apr 29;10(4):e0126409. doi: 10.1371/journal.pone.0126409 (PMC4414605; doi:10.1371/journal.pone.0126409)
Supplement: S1 Table — (PDF) [file pone.0126409.s005.pdf]

Supplementary Table 1. DIDA algorithm pseudo code.

```
Input: Set T of target sequences, set Q of query sequences
procedure DISTRIBUTE
    comp ← dfs(T)                                   ▷ identifying connected components
    comp_sorted ← qsort(comp)                       ▷ sorting connected components
    P ← best-fit-decreasing(comp_sorted)            ▷ partitioning sorted connected components
procedure INDEX
    for all p ∈ P in parallel do
        index_p ← build-index(p)                    ▷ constructing index for each partition
        store-index(index_p)                        ▷ storing index for alignment step
procedure DISPATCH
    for all p ∈ P in parallel do                    ▷ loading Bloom filter for each partition
        for all t ∈ p do
            for all b-mer ∈ t do
                insert(b-mer,BF[p])
    for all q ∈ Q do                                ▷ flowing queries through partitions
        for all p ∈ P in parallel do
            if contain(b-mer ∈ q,BF[p]) then
                dispatch(q,node_p)
procedure ALIGN
    for all p ∈ P in parallel do                    ▷ aligning queries against targets on all partitions
        receive(q, node_p)
        s ← align(q, index_p)
        send(s, node_merger)
procedure MERGE
    while receive(s, node_i) in parallel do         ▷ merging results from all partitions
        insert(s, priority-queue)
        s ← priority-queue.pop()
        write(s, samFile)
Output: File samFile
```
