# Supplementary material for: DIDA: Distributed Indexing Dispatched Alignment
Source: PLoS One. 2015 Apr 29;10(4):e0126409. doi: 10.1371/journal.pone.0126409 (PMC4414605; doi:10.1371/journal.pone.0126409)
Supplement: S2 Table — (PDF) [file pone.0126409.s006.pdf]

Supplementary Table 2. Exact numbers for *C. elegans* dataset - Fig. 2 in main text.

	time (sec)	mem (MB)	time (sec)	mem (MB)	time (sec)	mem (MB)	time (sec)	mem (MB)
				1-node				
	amap		bwa		bowtie		novoalign	
ind	30	1100	111	156	159	274	23	589
aln	2124	147	834	1400	1541	344	19648	554
total	2154	1100	945	156	1700	274	19671	589
				2-node				
	amap		bwa		bowtie		novoalign	
prt	1	16	1	16	1	16	1	16
ind	13	522	46	80	83	163	10	263
dsp	376	96	376	96	376	96	376	96
aln	875	73	281	842	628	248	5919	275
mrg	9	4	9	4	9	4	9	4
total	1261	522	667	80	1014	163	6305	263
				4-node				
	amap		bwa		bowtie		novoalign	
prt	1	16	1	16	1	16	1	16
ind	6	238	21	65	35	99	6	151
dsp	381	97	381	97	381	97	381	97
aln	491	39	172	804	335	217	4782	163
mrg	20	4	20	4	20	4	20	4
total	893	238	574	65	737	99	5184	151
				8-node				
	amap		bwa		bowtie		novoalign	
prt	1	16	1	16	1	16	1	16
ind	3	120	8	134	15	62	3	69
dsp	383	97	383	97	383	97	383	97
aln	296	21	99	784	168	195	4361	94
mrg	43	4	43	4	43	4	43	4
total	723	120	526	134	595	62	4788	69
				12-node				
	amap		bwa		bowtie		novoalign	
prt	1	16	1	16	1	16	1	16
ind	2	81	5	89	9	46	2	50
dsp	391	97	391	97	391	97	391	97
aln	249	16	96	783	150	190	4013	72
mrg	59	4	59	4	59	4	59	4
total	700	81	547	89	601	46	4464	50
