# Supplementary material for: DIDA: Distributed Indexing Dispatched Alignment
Source: PLoS One. 2015 Apr 29;10(4):e0126409. doi: 10.1371/journal.pone.0126409 (PMC4414605; doi:10.1371/journal.pone.0126409)
Supplement: S3 Table — (PDF) [file pone.0126409.s007.pdf]

Supplementary Table 3. Exact numbers for human draft assembly dataset - Fig. 3 in main text.

	time (min)	mem (MB)	time (min)	mem (MB)	time (min)	mem (MB)	time (min)	mem (MB)
1-node								
	amap		bwa		bowtie		novoalign	
ind	22	31000	73	4400	131	6100	8	9300
aln	630	4200	334	7100	1043	5300	59117	2600
total	652	31000	407	4400	1174	6100	59125	9300
2-node								
	amap		bwa		bowtie		novoalign	
prt	1	691	1	691	1	691	1	691
ind	9	15000	32	2200	54	2900	3	4100
dsp	100	2600	100	2600	100	2600	100	2600
aln	363		145		502		35619	
mrg	8	12	8	12	8	12	8	12
total	472	15000	254	2200	611	2900	35728	4100
4-node								
	amap		bwa		bowtie		novoalign	
prt	1	691	1	691	1	691	1	691
ind	4	8100	14	1100	29	1600	3	3700
dsp	101	2600	101	2600	101	2600	101	2600
aln	232	1510	105	2900	382	2300	23200	3500
mrg	9	12	9	12	9	12	9	12
total	343	8100	216	1100	493	1600	23311	3700
8-node								
	amap		bwa		bowtie		novoalign	
prt	1	691	1	691	1	691	1	691
ind	2	4100	7	559	13	977	2	2100
dsp	102	3300	102	3300	102	3300	102	3300
aln	130	387	68	1980	248	1660	17362	3100
mrg	20	12	20	12	20	12	20	12
total	253	4100	191	559	371	977	17485	2100
12-node								
	amap		bwa		bowtie		novoalign	
prt	1	691	1	691	1	691	1	691
ind	2	2700	5	372	8	590	1	1200
dsp	104	2600	104	2600	104	2600	104	2600
aln	81	387	47	1500	162	696	13007	2400
mrg	23	12	23	12	23	12	23	12
total	210	2700	181	372	296	590	13141	1200
