# Supplementary material for: DIDA: Distributed Indexing Dispatched Alignment
Source: PLoS One. 2015 Apr 29;10(4):e0126409. doi: 10.1371/journal.pone.0126409 (PMC4414605; doi:10.1371/journal.pone.0126409)
Supplement: S4 Table — (PDF) [file pone.0126409.s008.pdf]

Supplementary Table 4. Exact numbers for human reference genome (hg19) dataset.

	time (min)	mem (MB)	time (min)	mem (MB)	time (min)	mem (MB)
			1-node			
	amap		bwa		bowtie	
ind	21	33823	76	4709	129	5528
aln	423		303		867	
total	444	33823	379	4709	996	5528
			2-node			
	amap		bwa		bowtie	
prt	1		1		1	
ind	8	16911	33	2354	67	3042
dsp	103		103		103	
aln	212		143		401	
mrg	7		7		7	
total	323	16911	254	2354	512	3042
			4-node			
	amap		bwa		bowtie	
prt	1		1		1	
ind	3	8455	15	1177	26	1417
dsp	99		99		99	
aln	123		96		243	
mrg	9		9		9	
total	232	8455	205	1177	352	1417
			8-node			
	amap		bwa		bowtie	
prt	1		1		1	
ind	2	4227	7	588	11	667
dsp	93		93		93	
aln	63		61		144	
mrg	16		16		16	
total	173	4227	171	588	254	667
			12-node			
	amap		bwa		bowtie	
prt	1		1		1	
ind	1	3170	5	441	7	495
dsp	94		94		94	
aln	46		50		112	
mrg	19		19		19	
total	160	3170	164	441	226	495
