# Supplementary material for: DIDA: Distributed Indexing Dispatched Alignment
Source: PLoS One. 2015 Apr 29;10(4):e0126409. doi: 10.1371/journal.pone.0126409 (PMC4414605; doi:10.1371/journal.pone.0126409)
Supplement: S5 Table — (PDF) [file pone.0126409.s009.pdf]

Supplementary Table 5. Exact numbers for *Picea glauca* dataset.

	2-node		4-node		8-node		12-node	
	time (min)	mem (GB)	time (min)	mem (GB)	time (min)	mem (GB)	time (min)	mem (GB)
prt	10		10		10		10	
ind	207	184	68	81	28	45	17	31
dsp	341		210		164		161	
aln	829		584		438		370	
mrg	22		24		27		34	
total	1201	184	827	81	638	45	574	31
